# Do mechanisms matter? Comparing cancer treatment strategies across mathematical models and outcome objectives

**DOI:** 10.3934/mbe.2021315

**Published:** 2021-07-21

**Authors:** Cassidy K. Buhler, Rebecca S. Terry, Kathryn G. Link, Frederick R. Adler

**Affiliations:** 1 Department of Decision Sciences and MIS, Drexel University, 3220 Market St, Philadelphia, PA 19104, USA; 2 Department of Mathematics, University of Utah, 155 S 1400 E, Salt Lake City, UT 84112, USA; 3 Department of Mathematics, Computer Science and Statistics, St. Lawrence University, 23 Romoda Drive, Canton, NY 13617, USA; 4 Department of Mathematics, University of California, Davis, One Shields Avenue, CA 95616, USA; 5 School of Biological Sciences, University of Utah, 257 S 1400 E, Salt Lake City, UT 84112, USA

**Keywords:** adaptive therapy, cancer ecology, mathematical model, competition, Allee effect, androgen dynamics

## Abstract

When eradication is impossible, cancer treatment aims to delay the emergence of resistance while minimizing cancer burden and treatment. Adaptive therapies may achieve these aims, with success based on three assumptions: resistance is costly, sensitive cells compete with resistant cells, and therapy reduces the population of sensitive cells. We use a range of mathematical models and treatment strategies to investigate the tradeoff between controlling cell populations and delaying the emergence of resistance. These models extend game theoretic and competition models with four additional components: 1) an Allee effect where cell populations grow more slowly at low population sizes, 2) healthy cells that compete with cancer cells, 3) immune cells that suppress cancer cells, and 4) resource competition for a growth factor like androgen. In comparing maximum tolerable dose, intermittent treatment, and adaptive therapy strategies, no therapeutic choice robustly breaks the three-way tradeoff among the three therapeutic aims. Almost all models show a tight tradeoff between time to emergence of resistant cells and cancer cell burden, with intermittent and adaptive therapies following identical curves. For most models, some adaptive therapies delay overall tumor growth more than intermittent therapies, but at the cost of higher cell populations. The Allee effect breaks these relationships, with some adaptive therapies performing poorly due to their failure to treat sufficiently to drive populations below the threshold. When eradication is impossible, no treatment can simultaneously delay emergence of resistance, limit total cancer cell numbers, and minimize treatment. Simple mathematical models can play a role in designing the next generation of therapies that balance these competing objectives.

## 1. Introduction

Treatment that delays or prevents the emergence of resistance can control cancers, potentially indefinitely, and provides a suitable strategy when eradication is impossible [1]. As with bacterial resistance to antibiotics or herbivore resistance to pesticides, high levels of treatment can lead to emergence of resistant strains that had been controlled by a combination of costs of resistance and competition with susceptible strains [2]. Adaptive therapies, where cessation of treatment precedes loss of efficacy, have been proposed as a way to delay emergence of resistance, an approach supported by mathematical modeling, laboratory experiments, and some preliminary results of clinical trials [3–5]. Modeling has played a key role in evaluating therapeutic timing, often providing evidence that reduced doses with treatment holidays can provide longer-term control [6].

The effectiveness of adaptive therapy depends on three assumptions: resistance is costly, resistant cells can be suppressed by competition with sensitive cells, and therapy reduces the population of sensitive cells. Under these assumptions, mathematical models have provided some support for adaptive therapy [5, 7]. Our goal here is to investigate a wider range of mathematical models to better understand the role of these assumptions and of particular modeling choices in shaping the tradeoff between controlling cell populations and delaying the emergence of resistance. However, even for a relatively simple and well-understood cancer like prostate cancer, the mechanisms delaying resistance are not fully known. In particular, neither the role of costs nor that of competition with susceptible cell lineages has been clearly established.

For our range of models, we compare three strategies:

1. Maximum Tolerable Dose (MTD): a constant high dose determined by side effects.
2. Intermittent: a periodic scheduled dose with treatment holidays [8]. We here follow more common usage, rather than metronomic therapy, as recently reviewed [9].
3. Adaptive: initiation and termination of treatment based on the status of individual patient biomarkers, often with much earlier cessation of treatment than in intermittent strategies.

We begin by examining the original model by Zhang *et al* (2017) in more depth, and looking at a wider range of therapy strategies. Our central result is that all therapies follow the same tradeoff between total cancer cell burden and time to emergence of resistance.

To investigate a wider range of models, we first place the original model into a broader framework of game theoretic and competition models. We extend these in four ways that could alter responses to therapy.

- **Allee effect**: Control of resistance solely by competition with susceptible cell populations leads logically to the tradeoff between cancer cell burden and time to emergence of resistance. Inclusion of an Allee effect, where cell populations grow more slowly at low population sizes [10], could break that tradeoff.
- **Healthy cells**: Cancer cells compete both with each other and with unmutated cells [11] that respond differently to therapy and could alter the response to different therapeutic regimes.
- **Immune response**: Apparent competition, whereby species interact not through competition for space or resources, but as mediated by predators [12] or an immune response, could alter responses to therapy by introducing a delay and through their own responses to treatment [13, 14].
- **Resource competition:** Like competition mediated through the immune system, explicit resource competition models introduce delays mediated through the dynamics of depletable factors. Models include consumer-resource dynamics [15–17] and more mechanistic models of androgen dynamics [18–20].

After summarizing the original model from [5], we present the alternative models, provide parameter values that scale dynamics to be comparable to the original, derive analytical results on the simplest of these to illustrate tradeoffs, and test the three treatment strategies. We hypothesize that intermittent and adaptive therapy will produce similar results in all cases, with the exception of the Allee effect where the rapid cessation of treatment could allow cancers to escape.

## 2. Materials and methods

### 2.1. The basic Zhang model (Zh)

We begin with the model published by Zhang et al (2017). This Lotka-Volterra model has three competing cell types, which we reletter for consistency with our later models: S represents the population of androgen-dependent cells that are sensitive to treatment, P the population of androgen-producing cells and R the population of androgen-independent cells that are resistant to treatment $\left(x_{1},x_{2}\right.$ and $x_{3}$ respectively in the original).

(2.1) $$\frac{dS}{dt}\;=r_{S}\left(1-\frac{a_{SS}S+a_{SP}P+a_{SR}R}{K_{S}}\right)S\;\frac{dP}{dt}\;=r_{P}\left(1-\frac{a_{PS}S+a_{PP}P+a_{PR}R}{K_{P}}\right)P\;\frac{dR}{dt}\;=r_{R}\left(1-\frac{a_{RS}S+a_{RP}P+a_{RR}R}{K_{R}}\right)R.$$

Each cell type has an intrinsic per cell growth rate r and carrying capacity K. The carrying capacity for S cells is assumed to be proportional to P. The competition coefficients a represent pairwise competitive effects between cells. Adaptive therapy is based on prostate specific antigen (PSA) dynamics described by

(2.2) $$\frac{d\text{PSA}}{dt}=(S+P+R)-0.5\text{PSA}$$

where 0.5 represents the rate of PSA decay. This is much faster than the other time scales in the model, making PSA almost exactly proportional to total cell numbers.

This model simulates therapy by reducing the carrying capacity of S and P. More specifically, $K_{P}$ is reduced by a factor of 100, and the carrying capacity of S is changed from 1.5P to 0.5P. Treatment thus results in an extremely rapid drop in these two populations. Treatment has no effect on R cells which are thus released from the competitive suppression created by their smaller carrying capacity, and grow quickly until therapy is stopped. The full set of parameter values for our main simulations are given in Table 1 based on representative patient #1 [5].

This model includes the complexity of testosterone-producing (P) cells in addition to competition of sensitive and resistant cells. To test whether P cells are essential to the result, we build a simpler version of the model (Zs) with two cell types, and implement therapy by directly reducing the carrying capacity of the sensitive cells, with equations

(2.3) $$\frac{dS}{dt}\;=r_{S}\left(1-\frac{a_{SS}S+a_{SR}R}{K_{S}}\right)S\;\frac{dR}{dt}\;=r_{R}\left(1-\frac{a_{RS}S+a_{RR}R}{K_{R}}\right)R.$$

We use the same parameters as for the full model, except that $K_{S}=1.5\times 10^{4}$ in the absence of therapy and 50 with therapy.

### 2.2. General model framework

To capture the key assumptions of this model and examine the conditions that lead to success of adaptive therapy, we consider the following general framework of interaction between sensitive cells S and resistant cells R, and an additional variable or variables X:

(2.4) $$\frac{dS}{dt}\;=r_{S}(S,R,X,u)S-\delta_{S}(u)S\;\frac{dR}{dt}\;=r_{R}(S,R,X,u)R-\delta_{R}R\;\frac{dX}{dt}\;=f(S,R,X,u).$$

The additional dimension X represents androgen-producing cells in the Zhang model, but could also be healthy cells, androgen, another resource or growth factor, or an immune response. The variable u represents treatment, which will be a function of time for intermittent and adaptive therapy. The functions $r_{R}$ and $r_{S}$ describe growth as functions of population size to model competition, of X to capture the tumor microenvironment via use of resources or immune attack, and of u to represent the effects of treatment. The death terms $\delta_{S}$ and $\delta_{R}$ are included to separate births and deaths, and as a place for treatment effects u, here restricted to sensitive cells.

As outputs, we solve for the time of two types of treatment failure:

- The time $T_{C}$ when the total cancer cell population exceeds some threshold $C_{crit}$,
- The time $T_{R}$ when the population of resistant cells exceeds some threshold $R_{\text{crit}\;}$.

As the costs, we also track two outputs:

- Mean cancer cell numbers,
- The fraction of time being treated.

We use mean cancer cell numbers, which effectively assumes that costs are linear in the number of cells, both for simplicity and because risks of mutation and metastasis will be proportional to the number of cells. We do not attempt optimal control analysis [21], and present as results tradeoffs between the times, treatment, and cancer burden, looking for conditions where maximizing time to emergence reduces both treatment and cancer burden.

#### 2.2.1. Game theoretic model (GT)

The simplest version of this framework uses game theoretic models that focus on how strategy frequencies depend on frequency-dependent fitness. A basic model with density dependence is given by

(2.5) $$\frac{dS}{dt}\;=r_{S}(u)\left(1-\frac{S+R}{K}\right)S-\delta_{S}S\;\frac{dR}{dt}\;=r_{R}\left(1-\frac{S+R}{K}\right)R-\delta_{R}R.$$

where C=S+R. We place treatment costs in the growth rate of S cells, and give both cell types the same carrying capacity and symmetric competitive effects.

#### 2.2.2. Lotka-Volterra model (LV)

Equation 2.5 is a the special case of a Lotka-Volterra model with equal competition coefficients. We here generalize to a model similar to equation 2.1, but with a more realistic approach to the effects of therapy and consequences of resistance.


(2.6) $$\frac{dS}{dt}\;=r_{S}\left(1-\frac{S+a_{SR}R}{K_{S}}\right)S-\delta_{S}(u)S\;\frac{dR}{dt}\;=r_{R}\left(1-\frac{a_{RS}S+R}{K_{R}}\right)R-\delta_{R}R.$$

Treatment increases the death rate $\delta_{S}$. We scale the competition coefficients describing the effect of each type on itself to 1, but can have asymmetric competitive effects and different carrying capacities for the two cell types.

#### 2.2.3. Allee Effect (AL)

The next model complements this framework with an Allee effect, whereby cancer cells grow more quickly when the population is above some threshold. We use the form

(2.7) $$\frac{dS}{dt}=r_{S}\left(\frac{S+R}{k_{a}+S+R}\left(1-b\right)+b\right)\left(1-\frac{S+a_{SR}R}{K_{S}}\right)S-\delta_{S}\left(u\right)S\;\frac{dR}{dt}=r_{R}\left(\frac{S+R}{k_{a}+S+R}\left(1-b\right)+b\right)\left(1-\frac{a_{RS}S+R}{K_{R}}\right)R-\delta_{R}R.$$

The parameter b scales the strength of the effect, with b=1 reducing to equation 2.6. Values of 0<b<1 create a weak Allee effect where populations grow more slowly when rare, and b<0 generate a strong Allee effect where populations decline when rare. The parameter $k_{a}$ scales the critical population size below which the Allee effect is strongest.

#### 2.2.4. Healthy Cells (HC)

As our first example of an additional dimension X, we consider interactions with healthy cells, denoted by H.

(2.8) $$\frac{dS}{dt}\;=r_{S}\left(1-\frac{S+a_{SR}R+a_{SH}H}{K_{S}}\right)S-\delta_{S}(u)S\;\frac{dR}{dt}\;=r_{R}\left(1-\frac{a_{RS}S+R+a_{RH}H}{K_{R}}\right)R-\delta_{R}R\;\frac{dH}{dt}\;=r_{H}\left(1-\frac{a_{HS}S+a_{HR}R+H}{K_{H}}\right)H-\delta_{H}H.$$

In this simple model, healthy cells are distinguished by their reduced growth and lack of sensitivity to treatment.

#### 2.2.5. Lottery model with cancer growth (LM)

Our other models include carrying capacities, which is unrealistic for cancers. To address this, we create an extended lottery model of competition for sites with healthy cells [22]. Assume that healthy tissue has K sites, with H occupied by healthy cells and the rest E empty. The healthy cells turn over at rate $\delta_{H}$, and replicate at rate $r_{H}$ but only into empty locations. Then

(2.9) $$\frac{dE}{dt}=\delta_{H}H-r_{H}\left(\frac{E}{E+H}\right)H$$

(2.10) $$\frac{dH}{dt}\;=-\delta_{H}H+r_{H}\left(\frac{E}{E+H}\right)H.$$

This model maintains a constant number of sites E+H=K, and we think of the equilibrium healthy cell population as corresponding to a physiological optimum.

Cancer cells can differ from healthy cells in several ways: they may replicate more quickly, reproduce into sites occupied by healthy cells, and reproduce into sites occupied by other cancer cells and increase the total cell population. With two cancer cell types S and R, we have a total cell population of N=H+S+R and assume that E=K-N if N<K and E=0 otherwise. The cells follow the equations

(2.11) $$\frac{dH}{dt}\;=-\delta_{H}H+r_{H}\frac{E}{N}H-\eta \left(\frac{H}{N}\right)\left(a_{SH}r_{S}S+a_{RH}r_{R}R\right)\;\frac{dS}{dt}\;=-\delta_{S}(u)S+r_{S}\left(\frac{S}{N}\right)\left(E+a_{SH}H+a_{SS}S+a_{SR}R\right)\;\frac{dR}{dt}=-\delta_{R}R+r_{R}\left(\frac{R}{N}\right)\left(E+a_{RH}H+a_{RS}S+a_{RR}R\right).$$

The competition coefficients a describe the probability that a cancer cell reproduces into an occupied site. If that site is occupied by a cancer cell, we assume that the total cell population will increase, and the parameter η represents the probability that a cancer cell kills a healthy cell that it overgrows.

#### 2.2.6. Immune response (IC)

A simple model of the immune system obeys

(2.12) $$\frac{dS}{dt}\;=r_{S}\left(1-\frac{S+a_{SR}R}{K_{S}}\right)S-\delta_{S}(u)S-\eta_{S}IS\;\frac{dR}{dt}\;=r_{R}\left(1-\frac{a_{RS}S+R}{K_{R}}\right)R-\delta_{R}R-\eta_{R}IR\;\frac{dI}{dt}\;=r_{I}\left(\beta_{S}S+\beta_{R}R\right)\left(1-\frac{I}{K_{I}}\right)I-\delta_{I}I.$$

We assume that immune cells are induced to replicate by presence of cancer cells, and with growth limited by a carrying capacity. Immune cells directly kill cancer cells at rates $\eta_{S}$ and $\eta_{R}$. The replication and death rates of immune cells could be altered by treatment or by the effect of treatment in priming the immune response, although we do not address these factors here.

### 2.3. Models with androgen dynamics

We consider a range of models that include androgen-dependent growth by sensitive cells, and study androgen deprivation therapies that reduce the supply of this resource.

#### 2.3.1. Resource competition (RC)

We adapt the basic resource competition model from population biology [23] by treating androgen as a resource. Upon activation, androgen receptor is translocated to the nucleus, and we take the subsequent chemical transformations that occur within the cell to mean that any androgen that is used is destroyed in the process [24, 25]. The model tracks the two cell types and the androgen level A with the equations

(2.13) $$\frac{dS}{dt}\;=r_{S}(A)S\left(1-\frac{S+R}{K_{S}}\right)-\delta_{S}S\;\frac{dR}{dt}\;=r_{R}(A)R\left(1-\frac{S+R}{K_{R}}\right)-\delta_{R}R\;\frac{dA}{dt}\;=\sigma_{A}(u)-\delta_{A}A-c_{S}AS-c_{R}AR$$

where we model competition as in the basic game theory model (GT). Growth rates depend on androgen levels, and androgen is supplied externally at rate $\sigma_{A}$ that is reduced by treatment, and used by other cells at rate $\delta_{A}$. For simplicity, we assume non-saturating per cell absorption rates, but saturating growth

$$r_{S}(A)=\epsilon_{S}\frac{c_{S}A}{k_{S}+c_{S}A},\;r_{R}(A)=\epsilon_{R}\frac{c_{R}A}{k_{R}+c_{R}A}.$$

The parameters $\epsilon_{S}$ and $\epsilon_{R}$ describe growth efficiency of the two types, and $k_{S}$ and $k_{R}$ the half-saturation constants of growth as a function of androgen uptake. Androgen deprivation treatment reduces the supply rate of androgen, and has a larger effect on susceptible cells if $k_{S}>k_{R}$ or $c_{S}<c_{R}$.

#### 2.3.2. Androgen Dynamics (A3)

These more detailed models include androgen-dependent (S), androgen-producing (P), and androgen-independent cells (R) along with explicit dynamics of production and use. The dynamics follow

(2.14) $$\frac{dS}{dt}\;=r_{S}\left(A_{S}\right)\left(1-\frac{S+P+R}{K_{S}}\right)S\;\frac{dP}{dt}\;=r_{P}\left(A_{P}\right)\left(1-\frac{S+P+R}{K_{P}}\right)P\;\frac{dR}{dt}\;=r_{R}\left(1-\frac{S+P+R}{K_{R}}\right)R$$

where the division rates of S and P cells depend on their intracellular androgen concentrations $A_{S}$ and $A_{P}$ respectively.

The androgen concentrations derive from an accounting of androgen production and diffusion

(2.15) $$\frac{dA_{S}}{dt}\;=\eta \left(A_{E}-A_{S}\right)-\mu A_{S}\;\frac{dA_{P}}{dt}\;=\rho +\eta \left(A_{E}-A_{P}\right)-\mu A_{P}\;\frac{dA_{E}}{dt}\;=\sigma +\eta P\left(A_{P}-A_{E}\right)+\eta S\left(A_{S}-A_{E}\right)-\delta_{A}A_{E.}$$

$A_{E}$ is the external androgen concentration, η is diffusion into and out of cells, μ is androgen use by cells, ρ is production by P, and σ is residual production outside the prostate. The equilibrium of the androgen system is

(2.16) $$A_{E}\;=\frac{\sigma +\rho P\frac{\eta }{\eta +\mu }}{\eta (P+S)\frac{\mu }{\eta +\mu }+\delta_{A}}\;A_{S}\;=\frac{\eta }{\eta +\mu }A_{E}\;A_{P}\;=\frac{\rho }{\eta +\mu }+A_{S}.$$

We assume that the dynamics of equation 2.15 are sufficiently fast to use these equilibrium values in equation 2.14.

#### 2.3.3. Androgen model without androgen-producing cells (A2)

For comparison with the two-dimensional models, we simplify the system to exclude testosterone-producing cells by setting P=0 in equation 2.14.

### 2.4. Parameter values

We choose parameter values to hit three targets.

1. Resistant cells do not invade without therapy
2. Sensitive cells grow to about 10,000 without therapy
3. Resistant cells invade with MTD therapy

We set initial conditions to S(0)=1000 and $R(0)=1.0\times 10^{-10}$. The threshold for R cell invasion is set to $R_{\text{crit}\;}=1.0\times 10^{-4}$ and for total cell numbers to $C_{crit}=0.5M_{\text{max}}$ where $M_{max}$ is the maximum PSA level (equal to the total cancer cell population) that occurs in 10,000 days in the absence of treatment. We assume that treatment increases the death rate of sensitive cells by a factor of 50 for the models without resources to match the strong effect of treatment in the original model [5] except for the Allee effect model where we use a factor of 10 to avoid driving the population below the threshold too quickly. For the models with explicit resources (RC, A2 and A3) we reduce resource availability by 90% as an upper bound of observed effects [26, 27]. For treatment, we run a range of intermittent therapies with periods $t_{P}$ ranging from 100 to 1000 days and treatment duration $t_{D}$ running from 10 to 400 days, constrained to $t_{D}<t_{P}$. Treatment begins at time $t_{P}-t_{D}$. To implement adaptive therapy, we compare the levels of PSA to two critical levels. Therapy turns on when the total cell population increases above a fraction $M_{hi}$ of $M_{max}$ ranging from 0.2 to 0.9, and turns off when the total cell population decreases below a fraction $M_{lo}$ of $M_{max}$ ranging from 0.1 to 0.8.

### 2.5. Derivation of the tradeoff curve

Consider the basic model with two competing types,

(2.17) $$\frac{dS}{dt}=r_{S}\left(1-\frac{a_{SS}S+a_{SR}R}{K_{S}\left(u\right)}\right)S\;\frac{dR}{dt}=r_{R}\left(1-\frac{a_{RS}S+a_{RR}R}{K_{R}}\right)R$$

where u represents the level of drug. If R is rare, then it will have a negligible effect on S which will follow its own dynamics with solution S(t). R will obey the linear equation

$$\frac{dR}{dt}=r_{R}\left(1-\frac{a_{RS}S}{K_{R}}\right)R,$$

Integrating,

$$R(t)=R(0)e^{r_{R}t\left(1-a_{RS}\overset{‾}{S}/K_{R}\right)}$$

where $\overset{‾}{S}$ is the mean of S from time 0 to t. We solve for $T_{R}$ with $R\left(T_{R}\right)=R_{\text{crit}\;}$, or

(2.18) $$T_{R}=\frac{1}{r_{R}}ln\left(\frac{R_{\text{crit}}}{R(0)}\right)/\left(1-\frac{a_{RS}\overset{‾}{S}}{K_{R}}\right).$$

Because the models differ in their details, we include this relationship by fitting the parameters a and b to the predicted linear relationship of $\overset{‾}{S}$ with $1/T_{R}$,

(2.19) $$\overset{‾}{S}=a+\frac{b}{T_{R}}.$$

### 2.6. Computational methods

We solve models with the package deSolve in R [28] as written with one exception. In equation 2.14, there are two regulation terms based on androgen and carrying capacity, and the models behave pathologically when both are negative. In this case, we use only the androgen-based growth term. To simulate adaptive therapy, we include the auxiliary equation

(2.20) $$\frac{dU}{dt}=r_{U}\left(M>M_{hi}M_{max}\right)\left(1-U\right)-r_{U}\left(M<M_{lo}M_{max}\right)U+\;0.1r_{U}\left(M<M_{hi}M_{max}\right)\left(M>M_{lo}M_{max}\right)(U>0)\left(U<10U_{\text{crit}}\right)\left(U-U_{\text{crit}}\right)$$

where M represents the level of the marker, like PSA, which is set equal to the total number of cancer cells. Therapy is on when U>ϵ and off when U<ϵ. Uincreases when the marker M is above the threshold to turn on (the fraction $M_{hi}$ of the maximum value $M_{max}$) and decreases otherwise. We adjusted the parameter values to $r_{U}=20.0,\epsilon =1.0\times 10^{-8}$ and $U_{\text{crit}}=10\epsilon$ in order to have therapy remain on until the value of U decreases below a fraction $M_{lo}$ of $M_{max}$.

## 3. Results

We simulate adaptive and intermittent therapy, including as special cases MTD and No Therapy, and record $T_{R}$, the time when R cells emerge $\left(R(t)>R_{\text{crit}}\right)$ and $T_{C}$, the time when the total cancer cell population exceeds its threshold of $C_{\text{crit}}$. At each of these times, we record the average cell population until that time, and the fraction of time under treatment.

Intermittent therapy and adaptive therapy each have two hyperparameters that control the timing of treatment. For intermittent therapy, they are treatment period and duration, while for adaptive therapy they are $M_{hi}$ and $M_{lo}$, the fractions of the maximum PSA where treatment turns on and off respectively. By varying these hyperparameters, we obtain the tradeoff curves relating time to escape, cell burden, and treatment burden.

**Figure 1.**
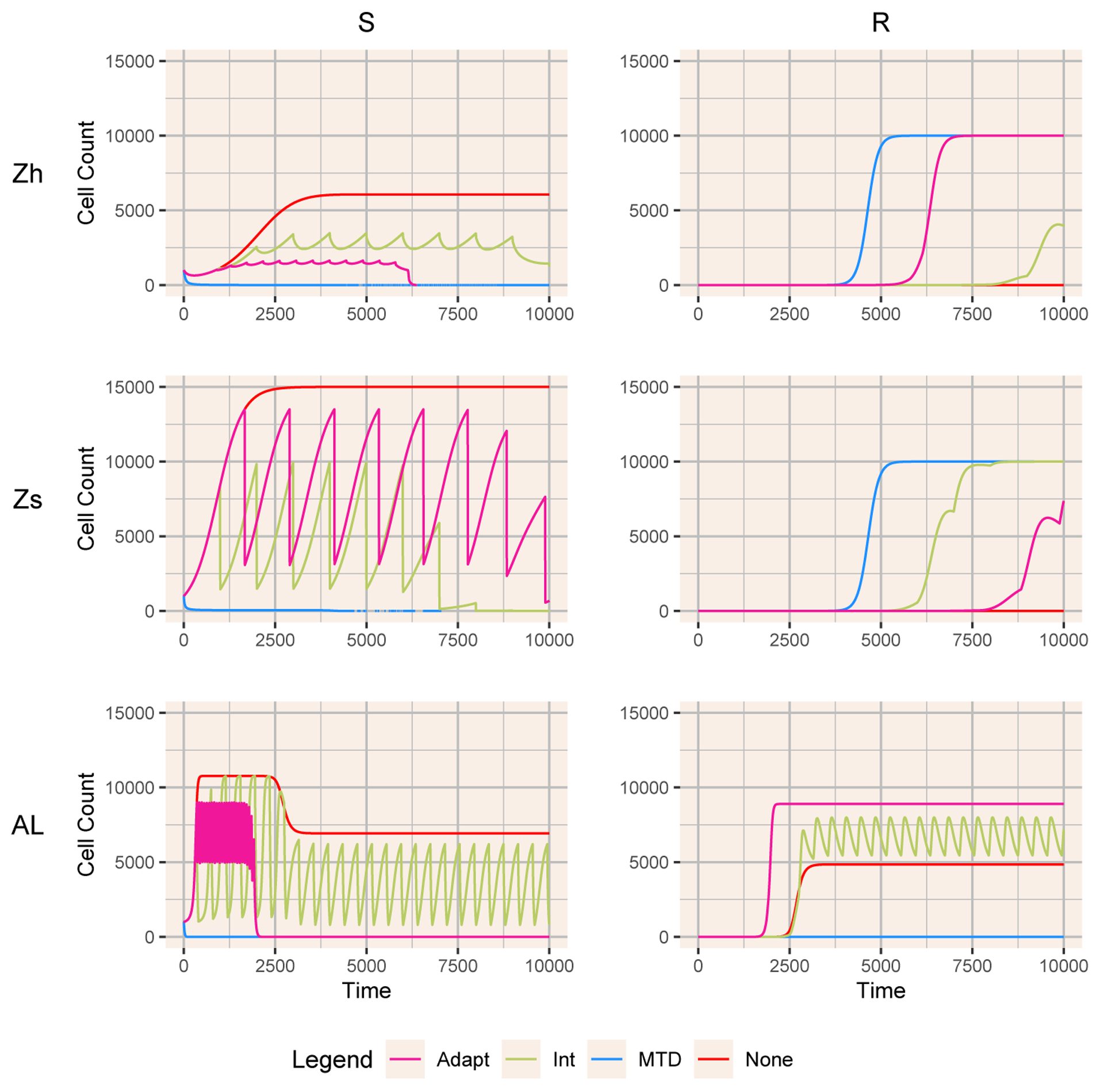
Dynamics of sensitive and resistant cells for three models (Zh, Zs, and AL) with the four treatments: MTD (blue), Adaptive (pink), Intermittent (green) and None (red). The adaptive therapy PSA bounds for each model are [0.6, 0.9], [0.8, 0.9], and [0.4, 0.5], respectively. The intermittent therapy treatment periods and treatment durations are [1000, 10], [1000, 10], and [400, 183.33].

To illustrate the dynamics, we show solutions of the equations for three of the models with representative parameters (Figure 1). In both the full and simplified Zhang models (Zh and Zs), MTD leads to the most rapid decline of sensitive cells and escape of resistant cells (blue curves), No Therapy leads to rapid increase of sensitive cells with no escape of resistant cells (red curves), and the greatest delay in emergence of resistance depending on the parameter choices. For the full range of hyperparameters, both intermittent and adaptive therapy generate repeated oscillations of sensitive cells that are eventually invaded and replaced by resistant cells. However, with Allee effect, the results are nearly the opposite. MTD drives the cancer cell population below the threshold and eliminates both sensitive and resistant cells. Resistance emerges most quickly with this choice of adaptive therapy, and intermittent therapy maintains both cell populations in a long-term oscillation.

**Figure 2.**
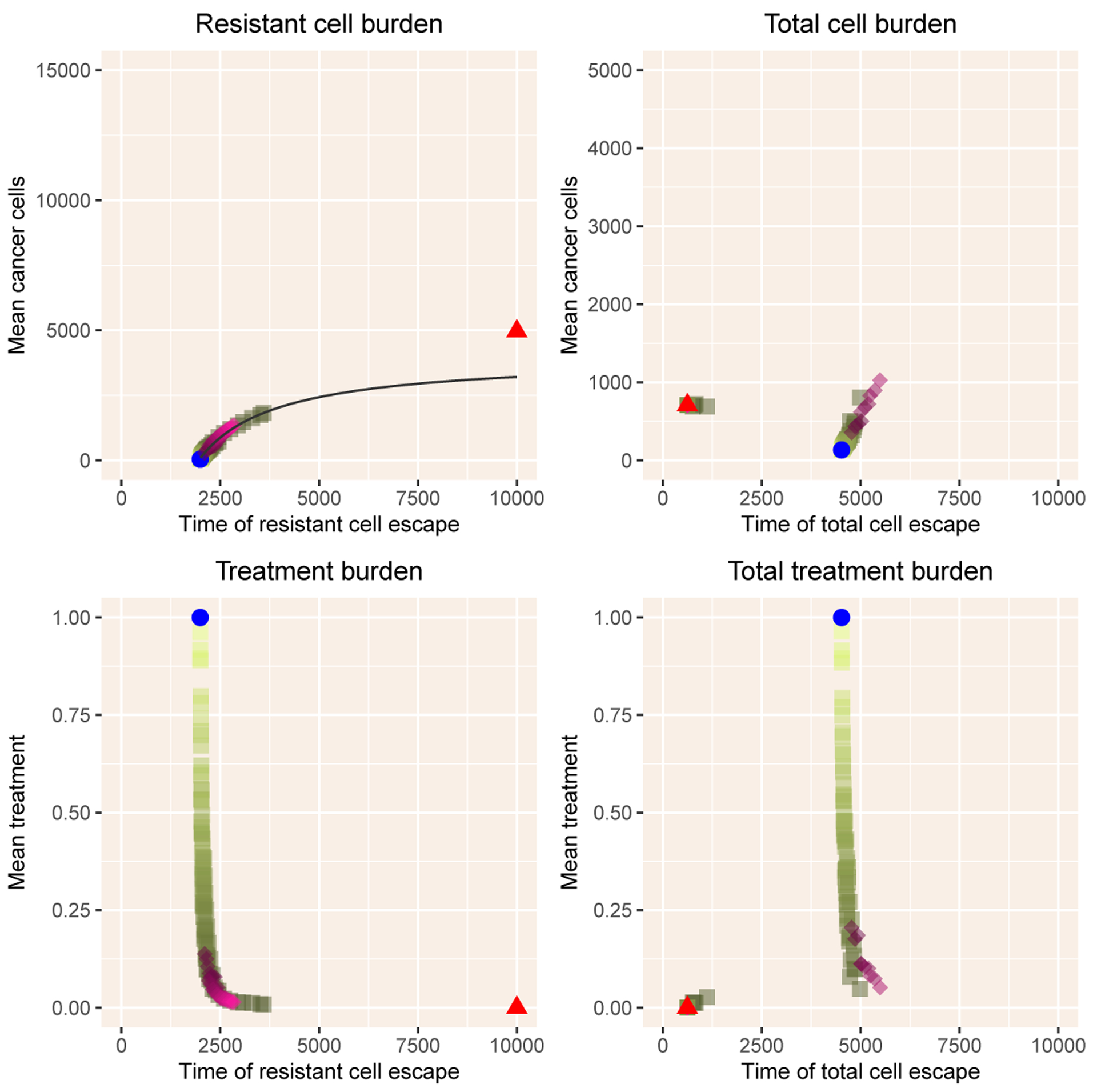
Summary of results with the Zhang model. The left column shows mean cancer cell burden and mean treatment burden as a function of the time of emergence $T_{R}$ of resistant cells above the critical value $R_{\text{crit}\;}$. The right column shows the same outputs as a function of the time $T_{C}$ of escape of cancer cells above the critical value $C_{\text{crit}}$. The blue dot indicates results with MTD and the red triangle results with No Therapy. The shades of green show intermittent therapy, with lighter shades indicating a higher fraction of time under treatment, the treatment duration divided by treatment period. The pink diamonds illustrate adaptive therapy, with darker shades indicating a lower value of $M_{lo}$, the threshold value for initiating therapy. The black line in the upper left panel is the curve in equation 2.19.

To summarize across the hyperparameters, we illustrate the relationships of time to escape of resistant and total cancer cells with mean cancer cell burden and mean tumor burden until that time beginning with the Zh model (Figure 2). Due to the simple linear dynamics of resistant cells when rare, intermittent and adaptive therapy follow the same tradeoff curves in relation to the time of escape of resistant cells (equation 2.19). MTD gives the endpoint of this curve, with the most rapid escape time, minimum cell burden and maximum treatment. The time to escape of the total cell population follows a more complex relationship, with some intermittent therapies acting much like No Therapy, with rapid cell population escape and a relatively high tumor burden. Adaptive therapy deviates only slightly from intermittent therapies, with a slight benefit of delaying escape at the cost of higher cell populations.

**Figure 3.**
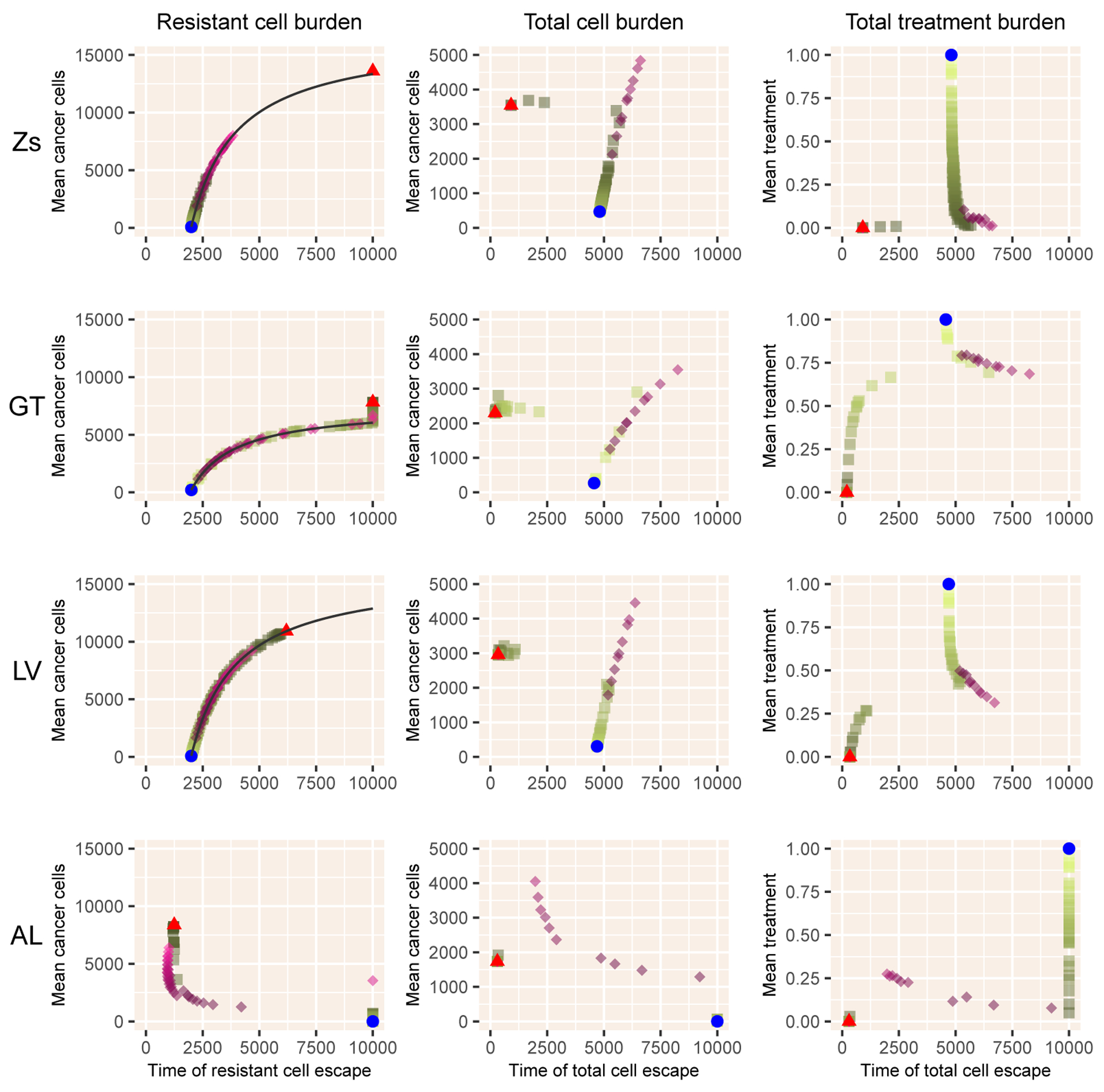
Results for the four models with sensitive and resistant cells only. Notation as in Figure 2.

The two-dimensional extensions follow similar dynamics with the exception of the Allee effect model AL (Figure 3). In the Zs, GT and LV models, the mean cancer cell burden follows the tradeoff curve (equation 2.19) for both intermittent and adaptive therapy. Overall, the two therapies behave similarly, although adaptive therapy can delay resistance at the cost of a slightly higher tumor burden. With the Allee effect, the results are quite different. MTD drives cells below the threshold, and prevents both resistant cells and total cells from reaching their thresholds. Adaptive therapy can behave quite poorly, leading to escape times nearly as short as those with No Therapy and with a high total cell burden.

**Figure 4.**
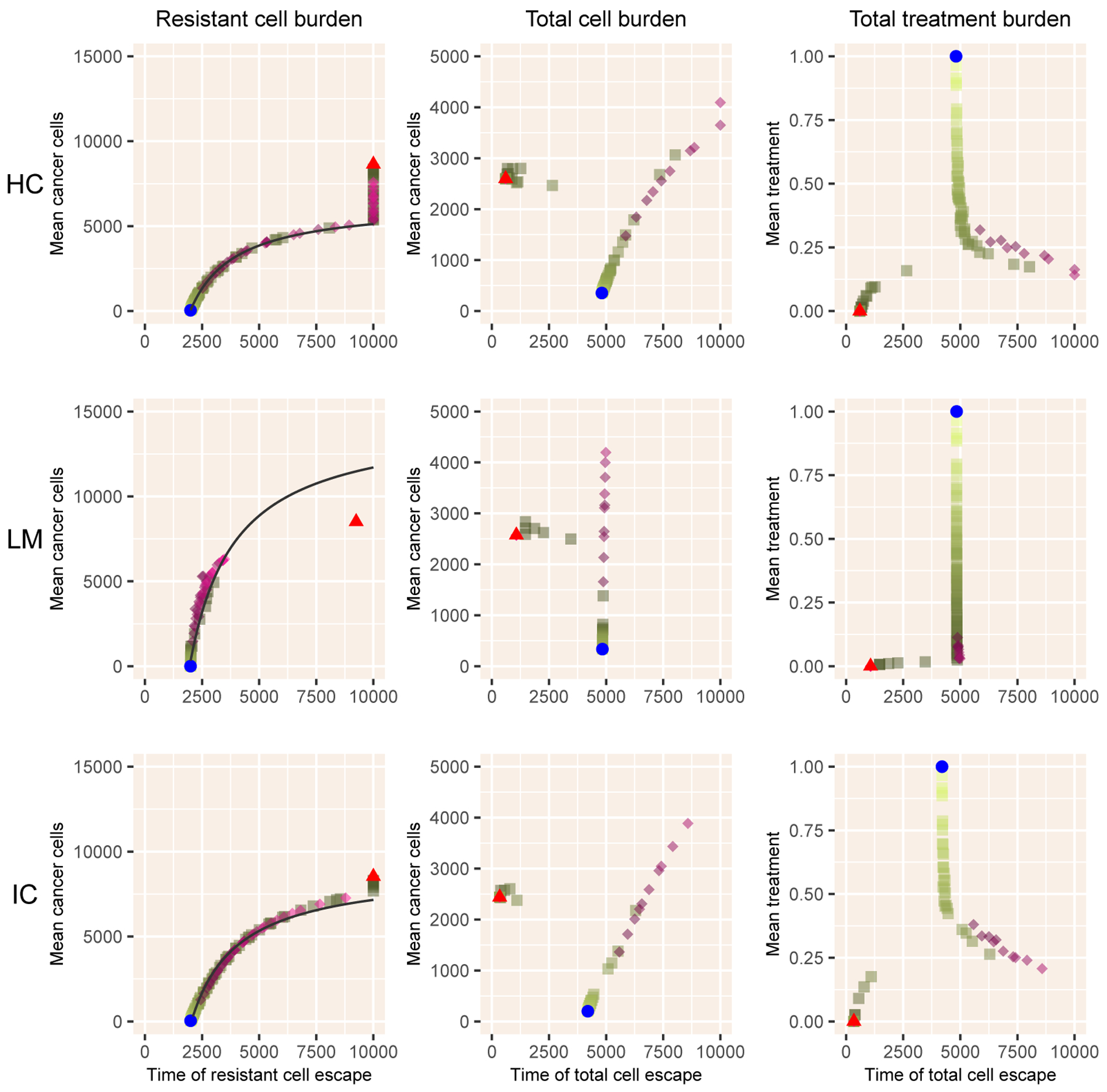
Results for the three models with sensitive and resistant cells plus an additional dimension. Notation as in Figure 2.

In Figure 4, we illustrate the results of the HC, LM, and IC models for which total cells are comprised of an additional cell type: healthy (HC, LM) or immune (IC). Despite their greater complexity, the results from these models also closely follow the predicted tradeoff (equation 2.19), although with a greater deviation for the lottery model (LM) that lacks a carrying capacity. As before, adaptive therapy largely overlaps with intermittent therapy, deviating in producing longer times to total cell escape at the expense of greater tumor cell burden, which is quite large for LM and IC models.

**Figure 5.**
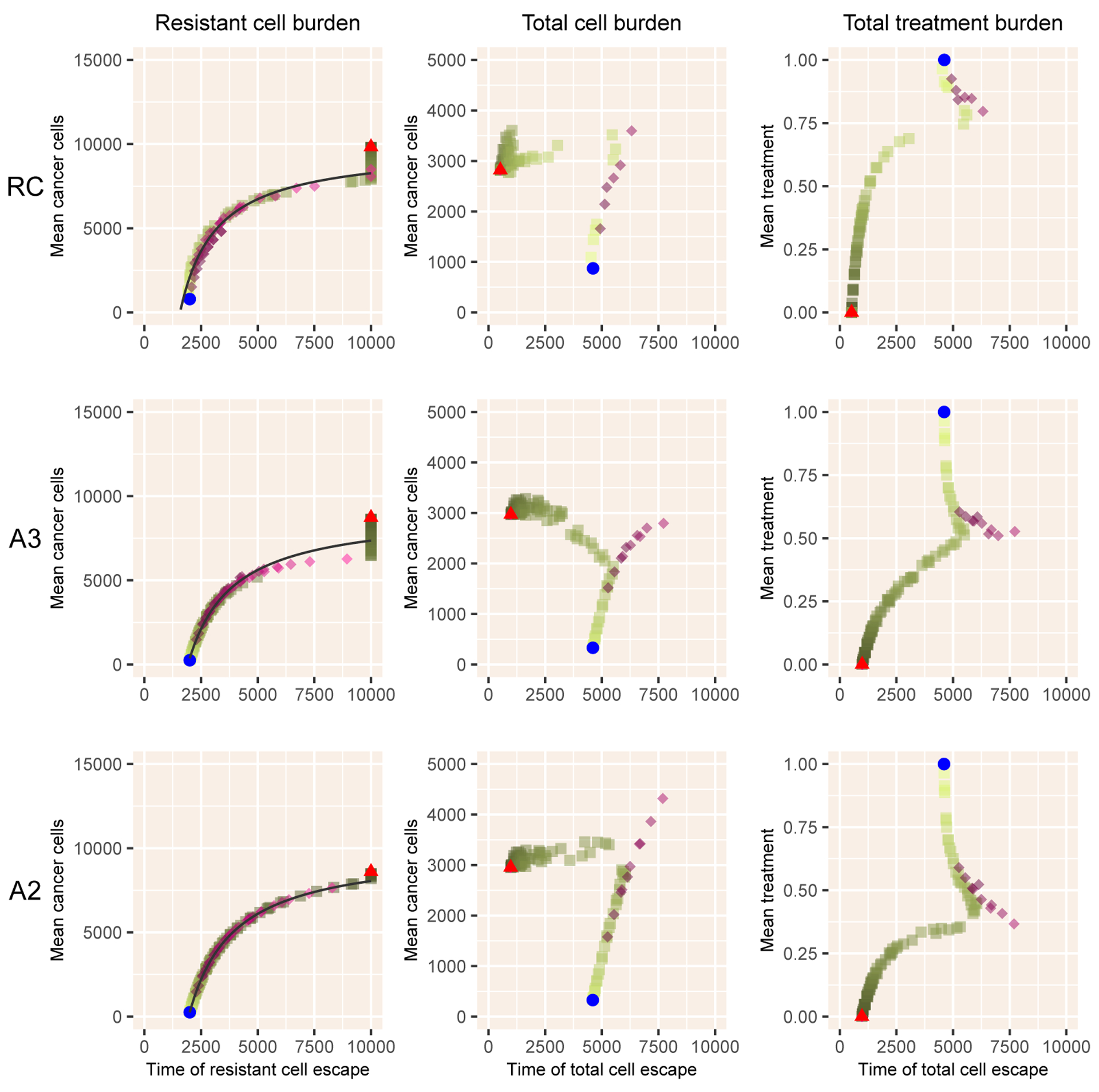
Results for the three models with androgen dynamics. Notation as in Figure 2.

The models with androgen dynamics, RC, A2, and A3, follow broadly similar patterns (Figure 5). The relationship of mean cancer burden with time to escape of resistant cells persists robustly. As before, adaptive therapy can deviate from intermittent therapy when considering time to escape of the total cancer cell number, with a more complex structure with two distinct branches evident with intermittent therapies that use a low level of treatment but not for any value of adaptive therapy.

With the exception of the success of MTD with a strong Allee effect, no universal therapy can achieve all three objectives of lowering mean treatment, delaying time to emergence of resistant cells, and reducing total tumor burden. All strategies follow the relationship of mean cancer burden with time to emergence of resistant cells, and the deviations between intermittent and adaptive therapies are relatively minor for time to escape of total cancer cells. The inclusion of androgen dynamics has the largest effect on whether intermittent, adaptive or MTD therapy has higher tumor cell burden with an earlier time to emergence of resistant cells.

## 4. Discussion

Adaptive therapy is based on three key assumptions: resistance is costly, resistant cells can be suppressed by competition with sensitive cells, and therapy is effective in reducing the population of sensitive cells. Without additional interactions, the only factor impeding resistant cells is competition with sensitive cells, leading to a tradeoff between reducing overall cancer burden and delaying emergence of resistance. We use a suite of simple models to find whether any of three therapy strategies can break this tradeoff: 1) constant therapy with maximum tolerable dose (MTD), 2) intermittent therapy on a fixed schedule, and 3) adaptive therapy on a patient specific schedule. We seek to test whether adaptive therapy is a feasible way to reduce overtreatment.

We have three key results. First, with the exception of models that include a strong Allee effect, all models closely follow a tradeoff curve between cancer cell burden and time to emergence of resistant cells, due to their suppression by sensitive cells. The Allee effect, meaning that cancer cell populations decline if they fall below some threshold, adds an additional type of control of resistant cells, and breaks the relationship. Second, again with the exception of models with an Allee effect, the tradeoff among time to total cancer cell number escape, average cancer cell burden, and total treatment, is similar but not identical over a range of intermittent and adaptive therapies. In most cases, some adaptive therapies do delay tumor growth, but at the cost of higher cell populations. With the Allee effect, some adaptive therapies perform quite poorly because the threshold for stopping therapy (a fraction $M_{lo}$ of $M_{\text{max}}$) is above the Allee threshold. Third, and most importantly, no therapeutic choice robustly breaks the three-way tradeoff among delaying emergence of resistance, delaying cancer growth, and minimizing treatment.

These simple models leave out many factors known to shape response to therapy. Most clearly, these models use ordinary differential equations that neglect spatial interactions known to be critical in shaping cell interactions and response to therapy [29–31]. The stochasticity neglected by differential equations would have the strongest effects when cell numbers are low, such as during the initial invasion of resistant cells or when populations are close to the threshold created by the Allee effect.

In addition, if resistance is induced by therapy rather than arising from mutations, resistance may be much more difficult to suppress [32]. Reversibility of these responses can create complex responses to therapeutic timing [33]. Much cancer resistance derives from phenotypic plasticity [34] that leads to more rapid emergence of resistance than the dynamics used here, and would require a different set of objective functions to evaluate.

Heterogeneity of cells with more than two states [35] can alter responses [30], and these states can be induced by a variety of intracellular changes including ABC transporter upregulation [36] and the evolution of mutation and genetic instability [37]. Androgen dynamic models begin to link intracellular with tissue-level models, but do not include specifics of pharmacokinetics and pharmacodynamics that generate differences among cells [36].

Our model of the immune system is highly simplified, and more realistic interactions could include the positive effects of the immune system on tumors [38], and additional thresholds, such as a lack of immune response to tumors below a particular size or an inability of the immune system to suppress tumors larger than some upper bound for control [39].

Use of multiple therapies, such as cytotoxic and cytostatic [40] can help create a double bind, such as between chemotherapeutic agents and those that attack glycolysis in hypoxic tumors [41] or force cancer into cycles of futile evolution [42]. The original paper [5] has been extended to include cells that are testosterone-independent and resistant to the chemotherapy docetaxel [43]. Oncolytic viruses interact with cells and the immune system with feedbacks that have only begun to be modeled [44]. Anti-angiogenic drugs have complex interaction with the dynamics of vasculature [14]. A modeling approach based on non-small cell lung cancer [45] that includes glycolytic cells and vascular overproducers, shows that adaptive therapy targeting glycolytic cells has potential to be effective [46].

We here examine only a few preset strategies, and optimization approaches could greatly refine these. The original model [5] itself has been studied this way [21]. Alternative models of prostate cancer [33] have been studied to retrospectively compute optimal treatments for 150 patients [47]. Using an alternative competitive framework with Gompertz growth, optimization of the dose and timing of treatment can maintain tumors below a tolerable size [48].

Optimization has been applied in many more complicated models, such as those including vasculature and the immune system and anti-angiogenic drugs and immunotherapy [49], and in models that include a continuum of internal resistance states and a population of healthy cells [40]. Building on this framework, a simplified two-state model identified strategies that give the full dose, then a smaller dose, and then a zero dose [50]. In a comparison of different treatment goals, a model of sensitive, damaged and resistant cells proposed to treat early to minimize integrated cell numbers, but early and late to minimize total cancer cell numbers at a fixed terminal time [51]. In a detailed model of colon cancer, [52] consider various dosing schedules to address the case where cancer cells can be in a quiescent state that is released by treatment of normal cells.

Any optimization requires choosing an objective function in a single ordered currency. Combining the measures used here (cancer cell burden, emergence of resistance, and costs of therapy) into a single currency would require sufficient clinical information to combine these into survivorship or quality-of-life adjusted years [53].

Applying even the simpler adaptive therapies here requires fitting to data on individual patients. A comparison of a simple model [33], a more complex model with basic androgen dynamics [20], and a detailed model of androgen dynamics [19] found that all fit data reasonably well, although with some exceptions [54]. Whether PSA dynamics alone are sufficient to resolve differences across patients, in particular patients who have different types of emerging resistance, seems unlikely, and methods almost certainly will need to be complemented with sequencing data [55]. If the models can be fit to the dynamics, adaptive therapies may be more robust to patient variability than prescribed timing of intermittent therapy.

Our evaluation shows that different objectives, delaying emergence of resistance, limiting total cancer cell numbers, and minimizing treatment, are likely to be related, and that no treatment can achieve all three. To address this, the choice of treatment strategy must be based on an objective function that weights different outcomes and patient goals, some of which are typically not included explicitly in mechanistic models, such as side effects [36].

Future therapies will need more sophisticated approaches that take into account multiple drug effects, differences among patients including increased clearance under low doses [6], and thinking that anticipates cancer responses [56, 57]. However, given the limited and noisy data we have on patients, we argue that the simple models and principles presented here will remain useful as we move toward the next generation of cancer therapy.

## Figures and Tables

**Table 1.** Parameter values used in the Zh model [5].

Parameter	Meaning	Value
*r_S_*, *r_P_*, *r_R_*	Growth rates of three cell types	0.0278; 0.0355; 0.0665
*K*_*S*_, *K*_*P*_, *K*_*R*_	Carrying capacities of cell types without treatment	1.5*P*; 1.0 × 10^4^; 1.0 × 10^4^
	with treatment	0.5*P*; 100; 1.0 × 10^4^
*a_S_*.	Effect of cell type S, P and R on *S* cells	1.0; 0.7; 0.8
*a*_*P*_.	Effect of cell type S, P and R on *P* cells	0.4; 1.0; 0.5
*a*_*R*_.	Effect of cell type S, P and R on *R* cells	0.6; 0.9; 1.0

**Table 2.** Models used in the paper. Each includes sensitive cells (S) and resistant cells (R).

Tag	Model Name	Additional Variables
Zh	Zhang model	*P* (androgen-producing cells)
Zs	Simplified Zhang model	
GT	Game theory	
LV	Lotka-Volterra	
AL	Allee effect	
HC	Healthy cells	*H* (healthy cells)
LM	Lottery model	*H* (healthy cells)
IC	Immune cells	*I* (immune cells)
RC	Resource competition	*P* (androgen-producing cells)
A3	Androgen dynamics	*P* (androgen-producing cells)
A2	Simplified androgen dynamics	

## Appendix A: Parameter values and justification

**Model Zh**: These parameters are chosen to match those in Zhang *et al* [5], but with time in units of days. $r_{S}=0.0278,r_{P}=0.0355,r_{R}=0.0665,K_{S}=1.5*P(0),K_{P}=10000,K_{R}=10000,a_{SS}=1,\;a_{SP}=0.7,a_{SR}=0.8,a_{PS}=0.4,a_{PP}=1,a_{PR}=0.5,a_{RS}=0.6,a_{RP}=0.9,a_{RR}=1,P(0)=300$. **Effects of therapy**: Reduce $K_{P}$ by 99% and reduce $K_{S}$ from $1.5K_{P}$ to $0.5K_{P}$.

**Model Zs**: These match those of Model Zh without the P cells. $r_{S}=0.0278,r_{R}=0.0665,K_{S}=15000,K_{R}=10000,a_{SS}=1,a_{SR}=0.8,a_{RS}=0.6,a_{RR}=1$. **Effects of therapy**: Reduce $K_{S}$ by 99%.

**Model GT**: $\;r_{S}=0.05,r_{R}=0.04,\delta_{S}=0.01,\delta_{R}=0.01,K=10000$. **Effects of therapy**: Reduce growth of S cells to 0, with R cells unaffected.

**Model LV**: $\;r_{S}=0.0278,r_{R}=0.0278,K_{S}=12000,K_{R}=10000,a_{SS}=1,a_{SR}=0.8,a_{RS}=0.6,\;a_{RR}=1,\delta_{S}=0.001,\delta_{R}=0.001$. **Effects of therapy**: Increase death rate of S cells by a factor of 50.

**Model AL**: $\;b=-0.1,k_{a}=5000,r_{S}=0.015,r_{R}=0.015,K_{S}=12000,K_{R}=10000,a_{SS}=1,\;a_{SR}=0.8,a_{RS}=0.6,a_{RR}=1,\delta_{S}=0.001,\delta_{R}=0.001$. **Effects of therapy**: Increase death rate of S cells by a factor of 10.

**Model HC**: $\;r_{S}=0.0278,r_{H}=0.0139,r_{R}=0.0555,K_{S}=12000,k_{H}=10000,K_{R}=10000,\;a_{SS}=1.0,a_{SH}=0.6,a_{SR}=1.0,a_{HS}=0.6,a_{HH}=1.0,a_{HR}=0.6,a_{RS}=1.0,a_{RH}=0.6,a_{RR}=1.0,\;\delta_{S}=0.001,\delta_{H}=0.001,\delta_{R}=0.001,H(0)=10000$. **Effects of therapy**: Increase death rate of S cells by a factor of 50.

**Model LM**: $\;r_{H}=0.0139,r_{S}=0.0278,r_{R}=0.05,K=10000,H(0)=10000,a_{SS}=0.008,\;a_{SH}=0.008,a_{SR}=0.008,a_{RS}=0.0,a_{RH}=0.008,a_{RR}=0.008\;\delta_{H}=0.001,\delta_{S}=0.001,\delta_{R}=0.001,\;\eta =0.3$. **Effects of therapy**: Increase death rate of S cells by a factor of 50.

**Model IC**: $c=0.4,r_{S}=0.0278,r_{I}=r_{S},r_{R}=r_{S},K_{S}=15000,K_{I}=1000,K_{R}=10000,a_{SS}=1,\;a_{SR}=0.8,a_{RS}=0.6,a_{RR}=1,\delta_{S}=0.001,\delta_{I}=0.001,\delta_{R}=0.001,\beta_{S}=2/K_{S},\beta_{R}=2/K_{S},\;\eta_{S}=c*\left(\left(r_{S}-\delta_{S}\right)/K_{I}\right),\eta_{R}=\eta_{S},I(0)=1000$. We give immune cells a carrying capacity of $K_{I}=1000$ for convenience. We set $\beta_{S}=\beta_{R}=2/K_{S}$ so that the immune system is strongly primed when cancer cell populations are large. We pick

$$\eta_{S}=\eta_{R}=c\frac{r_{S}-\delta_{S}}{K_{I}}$$

where c determines the strength of the immune response. **Effects of therapy**: Increase death rate of S cells by a factor of 50.

**Model RC**: $c_{S}=5e-5,c_{R}=c_{S},\sigma_{A}=50,\epsilon_{S}=0.01,\epsilon_{R}=0.015,K_{S}=15000,K_{R}=10000,\;k_{S}=0.005,k_{R}=0.1*k_{S},\delta_{S}=0.001,\delta_{R}=0.001,\delta_{A}=0.5,A(0)=100$. **Effects of therapy**: Reduce the resource supply rate $\sigma_{A}$ by 90%.

**Model A3**: $r_{0}=0.00208,s_{S}=0.0313,s_{P}=0.00581,r_{R}=0.00565,K_{S}=15000,K_{P}=10000,\;K_{R}=10000,\delta_{A}=6000,\rho =10,\mu =9,\sigma =10000,\eta =1,P(0)=1.0$. **Effects of therapy**: Reduce the resource supply rate σ and resource production rate ρ by 90%. We present the details of the derivation of these values in Appendix B.

Model A2: $\;r0=0.00208,sS=0.0313,r_{R}=0.00565,K_{S}=15000,K_{R}=10000,\delta_{A}=6000,\rho =10,\;\mu =9,\sigma =10000,\eta =1$. **Effects of therapy**: Reduce the androgen supply rate σ by 90%.

## Appendix B: Calculation of parameter values for Model A3

To find the parameter values that roughly match our targets, we aim to have

- S and P can coexist with total population of $1.0\times 10^{4}$,
- About 90% of androgen comes from P cells, and is used internally and fairly quickly,
- With treatment that reduces σ and ρ by 90%, populations decline quickly.

The key idea is that the growth functions take the form

$$r_{S}\left(A_{S}\right)=-r_{0}+s_{S}A_{S}\;r_{P}\left(A_{P}\right)=-r_{0}+s_{P}A_{P}$$

and must satisfy the conditions that $r_{S}\left(A_{S}^{*}\right)=\tilde{r_{S}}$ and $r_{P}\left(A_{P}^{*}\right)=\tilde{r_{P}}$ taken as the values from Model Zh. To find $r_{0}$, we choose a value of $A_{S,\text{ crit }}<A_{S}^{*}$ which is point of zero growth. We then find

$$\begin{array}{rr} r_{0} & \;=\tilde{r_{S}}\frac{A_{S,\text{ crit }}}{A_{S}^{*}-A_{S,\text{ crit }}}\\ s_{S} & \;=\frac{\tilde{r_{S}}}{A_{S}^{*}-A_{S,\text{ crit }}}. \end{array}$$

We can then find $A_{P\text{,crit }}$ by assuming that S and P cells have the same value of $r_{0}$ as

$$A_{P,\text{ crit }}\;=A_{P}^{*}\frac{r_{0}}{r_{0}+\tilde{r_{P}}}\;s_{P}\;=\frac{\left.\tilde{(}r_{P}\right)+r_{0}}{A_{P}^{*}}$$

The equilibrium equations for S and P take the form

$$0=\left(-r_{0}+s_{S}A_{S}\right)\left(1-\frac{S+P}{K_{S}}\right)\;0=\left(-r_{0}+s_{P}A_{P}\right)\left(1-\frac{S+P}{K_{P}}\right)$$

To coexist, we must have one type regulated by the carrying capacity and the other by androgen. Because $K_{P}<K_{S}$, P must be regulated by carrying capacity, so $S+P=K_{P}$. Then S will be regulated by androgen, meaning that $A_{S}=A_{S,\text{ crit }}$ at equilibrium. We have that

$$A_{S}\approx \frac{\sigma +\rho P\frac{\eta }{\eta +\mu }}{\mu K_{P}}=A_{S,\text{ crit }}$$

by setting $\delta_{A}=0$ and simplifying by using $S+P=K_{P}$. This will have a positive root for $P^{*}$ as long as $A_{S\text{,crit }}$ is sufficiently large. Otherwise, P will be excluded from the system because external production alone can maintain S at a high enough level to exclude it. The root is

$$P^{*}=\frac{\mu K_{P}A_{S,\text{ crit }}-\sigma }{\rho \frac{\eta }{\eta +\mu }}$$

We suppose η=1.0 and μ=9.0, meaning that most androgen is used internally and fairly quickly. Results are not sensitive to $\delta_{A}$ and we pick $\delta_{A}=6000.0$ for convenience. The value of ρ can be scaled out and we set ρ=10.0 for convenience. For 90% of androgen to come from P cells, σ=0.1⋅0.1ρP. To find equilibrium androgen values, we need equilibrium values of S and P. In the Zhang model with $a_{SP}=a_{PS}=1$, we have $P^{*}=2K_{P}/3=20000/3$ and $S^{*}=K_{P}/3=10000/3$. By picking σ=10000, and $A_{E}^{*}=0.511,A_{S}^{*}=0.0511,A_{P}^{*}=1.0511$. We assume that $K_{S}=15000$, and $K_{P}=K_{R}=10000$.
